# Contribution of Longitudinal Mobile Health Measures in the Dynamic Track of Patients With Major Depressive Disorder: Multiple Centers, Prospective Cohort Study Using Functional Data Analysis and Machine Learning

**DOI:** 10.2196/81397

**Published:** 2026-05-11

**Authors:** Rou Zhong, Nanxi Li, Le Xiao, Lei Feng, Yuan Feng, Gang Wang, Xuequan Zhu

**Affiliations:** 1 Beijing Key Laboratory of Intelligent Drug Research and Development for Mental Disorders, National Clinical Research Center for Mental Disorders, National Center for Mental Disorders, Beijing Anding Hospital, Capital Medical University, No. 5 Ankang Hutong, Xicheng District, Beijing, China, 86 15201109903; 2 Advanced Innovation Center for Human Brain Protection, Capital Medical University, Beijing, China

**Keywords:** major depressive disorder, mobile health, patient-reported outcome, digital phenotype, functional data analysis, machine learning

## Abstract

**Background:**

Continuous follow-up for patients with major depressive disorder (MDD) is essential for treatment decisions and a better prognosis. There remains limited evidence regarding the critical issue of depression variation trajectory prediction using mobile health (mHealth) measures. Moreover, the temporal dynamics of mHealth measures have not been fully modeled in previous studies, and the poor patient adherence to mHealth records poses great challenges to the dynamic feature modeling.

**Objective:**

This study aimed to examine the contribution of mHealth measures in predicting depression variation trajectory for patients with MDD, with full consideration of the temporal dynamics of mHealth measures.

**Methods:**

A total of 229 patients with MDD from a multiple-center, prospective cohort were included. A 12-week follow-up was conducted involving the collection of the Hamilton Depression Rating Scale (HAMD-17), along with patient-reported outcomes (Immediate Mood Scaler and Altman Self-Rating Mania Scale) via mobile devices and sleep duration through wearable wristbands. We used functional data analysis to extract dynamic features from the sparse mHealth records, rather than aggregating the data to a single scalar summary measure through collapsing over time. Subsequently, 3 machine learning models were applied to predict the depression variation trajectory classes based on the baseline characteristics and these extracted dynamic features.

**Results:**

Based on the variation of HAMD-17 scores within 12 weeks, the participants were labeled into 4 classes through the *k*-means algorithm. The classes included stable decline (n=93), fluctuate decline (n=44), fast decline (n=60), and delayed and fluctuate (n=32), in light of the shape of depression trajectories. With both baseline features and dynamic features of the mHealth measures, accuracy rates for the overall data were 54.35%, 60.87%, and 56.52%, for the stable decline patients were 78.95%, 84.21%, and 73.68%, for the nonstable decline patients were 59.26%, 62.96%, and 70.37% based on the 3 machine learning models, respectively. The results were significantly superior to the prediction obtained without mHealth measures (with an overall accuracy below 50%) and only showed a marginal reduction in accuracy relative to the ideal prediction with assessment obtained from clinical visits. Moreover, in the construction of the most accurate prediction model, dynamic features of the Immediate Mood Scaler, the Altman Self-Rating Mania Scale, and sleep duration emerged as the most influential predictors, ranking first, third, and fourth, respectively, in terms of their relative importance.

**Conclusions:**

Longitudinal mHealth measures show potential in depression variation trajectory monitoring for patients with MDD even under poor patient adherence. Our work provides practical help in alleviating the follow-up burden for patients with MDD and validates the effectiveness of mHealth measures in clinical applications.

## Introduction

Depression is a common mental disorder with a high lifetime prevalence, ranked as one of the most crucial causes of disease burden worldwide [1,2]. Despite the availability of evidence-based treatments, including antidepressants and psychotherapy, more than 60% of patients with major depressive disorder (MDD) exhibit inadequate response with their first treatment, often requiring either an alternative antidepressant or adjunctive treatment [3]. The early reduction of depressive symptoms observed in the first 2‐4 weeks of antidepressant treatment has been proposed as a potential predictor of treatment success, possibly reflecting psychological resilience in patients with MDD [4], and the predictive efficacy of early response varies across antidepressants. Leveraging time-varying individual characteristics, including demographics, clinical phenotypes, and social functioning [5], holds promise for refining treatment algorithms. An important question raised in this context is how to capture the temporal dynamics of depression severity, which remains a persistent challenge in both clinical practice and research.

It is burdensome for both patients and doctors to conduct continuous and frequent face-to-face psychometric evaluations. Fortunately, with the increasing use of smartphones and wearable devices in modern life, it has become possible to achieve real-time monitoring of physical activity, sleep, and self-assessment for patients with MDD through mobile health (mHealth) technologies [6-8]. The mHealth measures obtained from smartphones and wearable devices can be summarized into 2 types, which are self-reported data (patient-reported outcomes [PROs] such as questionnaire scores) and passively collected data (digital phenotype data such as sleep data, activity data, and phone usage data) [9]. Smartphones are superior in capturing mood changes, while wearable devices are effective in daily activity records [10]. The incorporation of records from both smartphones and wearable devices is beneficial for clinical evaluation and provides a new perspective for depression monitoring [7].

For the potential of self-reported data recorded by smartphone in the depressive symptoms monitoring, Torous et al [11] examined the correlation between the Patient Health Questionnaire-9 scores recorded by smartphone and paper for the patients with MDD, demonstrating the capability of digital depressive symptom monitoring. Moreover, Goltermann et al [9] also revealed the validity of smartphone-based monitoring of depressive symptoms through the analysis of the agreement between smartphone-based and non–smartphone-based assessments. These studies highlight the effectiveness of smartphone-based self-reported data in tracking depressive symptoms.

Digital phenotype data have been widely applied in mental health studies and have shown the ability to strengthen the management of depressive symptoms [12,13]. As sleep problems occur frequently in patients with MDD [14], an increasing number of studies focused on the relationship between remote sleep records and depression symptoms [15,16]. Moreover, Lim et al [17] used sleep-wake pattern data exclusively in the prediction of mood episodes. Further, compared with other types of digital phenotype data, sleep data are easier to collect and less affected by other interfering factors [18]. These findings revealed the potential of remote sleep records in enhancing depression monitoring and prediction.

As depressive symptoms are constantly evolving and show heterogeneity over time, capturing the temporal dynamics of depression severity is essential [19]. However, given the great importance of depression dynamics, studies on the prediction of depression variation trajectory through mHealth measures were scarce. Bai et al [20] discussed the prediction of mood swings for patients with MDD using passively collected data from smartphones and wearable devices. Price et al [21] used digital phenotype data in the prediction of depression symptom variability. The definition of depression variation was still vague in existing mHealth literature and lack intuitiveness and clinical significance. And there is a growing demand for ascertaining the contribution of mHealth measures in depression variation trajectory prediction.

Additionally, fusing the temporal dynamic features of smartphone- and wearable device–based data in the prediction of depression trajectory is also crucial due to the dynamic nature of personalized behaviors [22-24]. However, the records from smartphones and wearable devices are always sparse and irregular, owing to poor study adherence and unsatisfactory patient compliance [25-27]. Patients may accidentally switch off the app or forget to report their states. The sparsity of the mHealth records poses additional challenges to the capture of dynamic features for both self-reported data and digital phenotype data, which calls for the clinical application of novel statistical methods.

**Figure 1.**
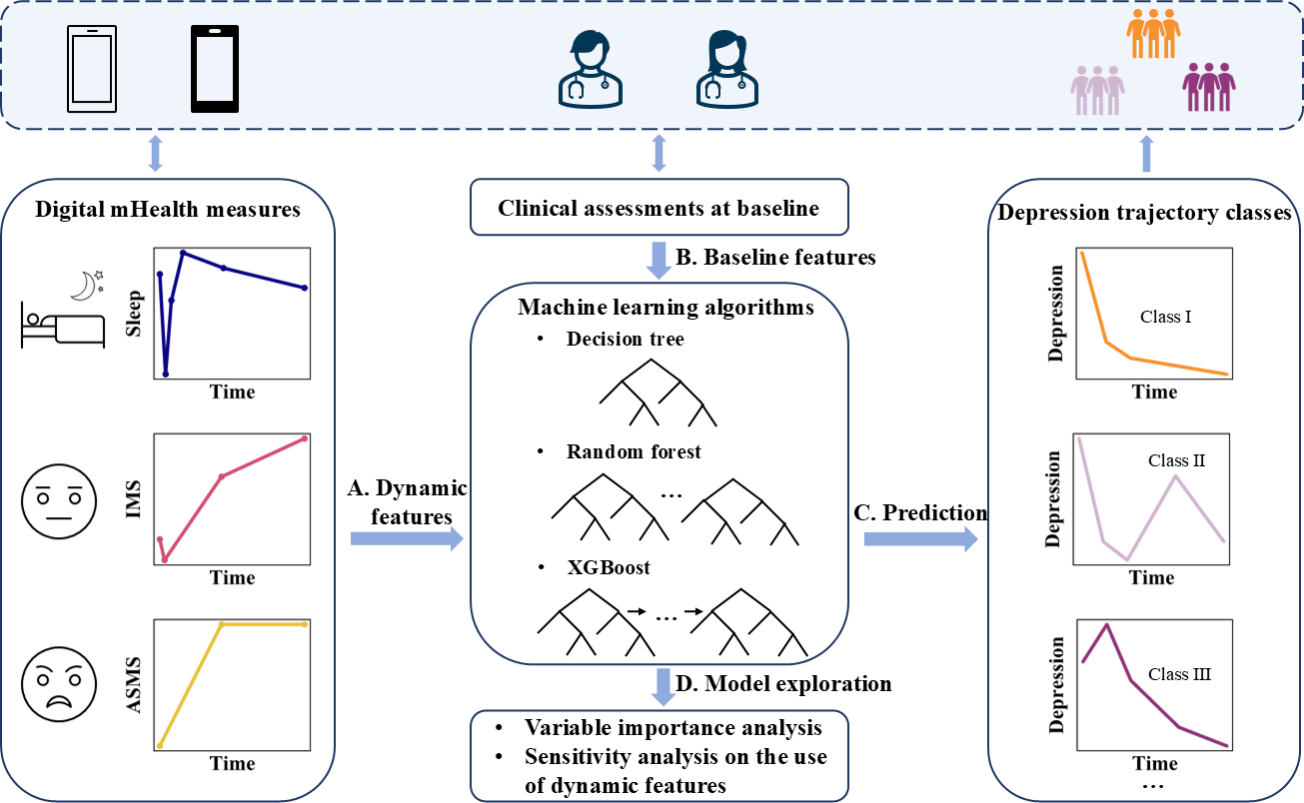
Study framework for detecting the contribution of longitudinal mHealth measures in depression variation trajectory prediction. ASMS: Altman Self-Rating Mania Scale; IMS: Immediate Mood Scaler; mHealth: mobile health.

The primary objective of this study is to examine the contribution of longitudinal mHealth measures in predicting depression variation trajectory for patients with MDD. By applying a functional data analysis method to the longitudinal records of sleep duration, Immediate Mood Scaler (IMS) scores, and Altman Self-Rating Mania Scale (ASMS) scores, the dynamic features of mHealth measures (including both PROs and digital phenotypes) were obtained (Figure 1A). The depression trajectory class was predicted (Figure 1C) based on the dynamic features of mHealth measures and clinical assessments at baseline (Figure 1B) through machine learning algorithms. The investigation of classification accuracy with and without the longitudinal records of PROs and digital phenotypes, as well as the variable importance analysis and sensitivity analysis (Figure 1D), enables the detection for the contribution of the dynamic features of mHealth measures in depression variation trajectory prediction for patients with MDD.

## Methods

### Ethical Considerations

This study used data from an epidemiological, noninterventional, cohort study conducted between February 2019 and April 2020 at 4 psychiatric hospitals or units in general hospitals in China. The study was approved by the Independent Medical Ethics Committee Board of Beijing Anding Hospital and the other 3 sites (ethical approval no. 2018-119-201917FS-2). All patients provided written informed consent to participate in the study. To ensure confidentiality, each participant was assigned an identification number. Moreover, participants received ¥100 (US $15.5) for each follow-up visit.

### Participants

Study inclusion and exclusion criteria have been reported [20]. Generally, inclusion criteria for the study included outpatients (1) aged 18‐60 years and (2) diagnosed with MDD according to the *Diagnostic and Statistical Manual of Mental Disorders* (fourth edition) criteria. Exclusion criteria included patients who had axis I primary psychiatric diagnosis other than MDD or had substance abuse. Patients in this cohort were followed up at 2, 4, 8, and 12 weeks.

### Measures

#### Demographics and Clinical Characteristics

The participants’ basic social-demographic and clinical characteristics were collected through the structured questionnaire designed for this study.

#### Clinical-Rated Outcomes

The Hamilton Depression Rating Scale (HAMD-17) [28] was used to measure depressive symptoms. The total HAMD-17 score ranges from 0 to 52, with a higher score indicating greater severity of depression.

The Hamilton Anxiety Scale (HAMA) [29] was used to measure anxiety symptoms. The total HAMA score ranges from 0 to 56, and a higher score indicates the severity of anxiety.

The Sheehan Disability Scale [30] was used to measure the social functional impairment due to MDD that interferes with work/school, social life/leisure activities, and family life/home responsibilities. The participants are invited to rate the extent to which each domain is impaired by their symptoms using a 10-point scale (0=not at all impaired, 1‐3=mildly impaired, 4‐6=moderately impaired, 7‐9=markedly impaired, and 10=extremely impaired).

#### Patient-Reported Outcomes

The Chinese version of the IMS was used to assess current mood symptoms [31,32]. It is composed of 22 questions about the respondent’s state of mood. Each item was rated on a 7-point scale with complementary antonyms positioned at opposite ends. A higher summed score of the 22 items reflects a higher level of severity of the patient’s depressive symptoms. Upon enrollment, participants were instructed to complete the IMS scale on their smartphones on a daily basis.

The ASMS was administered as a self-assessment tool to evaluate the intensity of manic symptoms [33]. The ASMS comprises five key symptom domains: (1) elevated mood, (2) heightened self-esteem, (3) diminished need for sleep, (4) rapid or pressured speech, and (5) psychomotor agitation. Patients rated each item by choosing from 5 response options, ranging from 0 (symptom not present) to 4 (highly severe). Participants were instructed to complete weekly administrations of the ASMS using their smartphones.

#### Digital Phenotype

Participants were instructed and trained to wear wristbands during sleep for sleep-related data collection during the 12-week follow-up. The data collected would first be stored locally and uploaded to our server when the user connects with the wristband using the Mood Mirror app [20]. Key sleep characteristics, particularly total sleep duration and slow-wave sleep time, were systematically extracted and incorporated into the analytical model.

### Statistical Analysis

#### Clustering Analysis

In this paper, we mainly focused on the variation of depression severity at 5 consecutive visits within 12 weeks. Therefore, the class labels for the patients were expected to be related to the variation of depression severity. To determine the class label for each patient, the *k*-means clustering algorithm was used. The *k*-means clustering algorithm is an unsupervised learning method that can divide samples into *k* clusters based on the cluster means. We computed the difference of HAMD-17 scores between each adjacent visit (week 2 to week 0, week 4 to week 2, week 8 to week 4, and week 12 to week 8) for each patient and input these features into the clustering analysis. That means patients who had a similar changing trend of HAMD-17 score would be assigned to the same cluster. We implemented the *k*-means algorithm through the kmeans function in R statistical software, version 4.4.1. The number of clusters was selected by considering both the silhouette coefficients and the clinical significance of the clustering results. The silhouette coefficients were computed by the silhouette function of the cluster R package, version 2.1.6. After comparing the results obtained by *k*=2,3,4,5, we chose the cluster number as 4 and further defined the class labels as stable decline, fluctuate decline, fast decline, and delayed and fluctuate.

#### Feature Extraction by Functional Principal Component Analysis

Within the 12 weeks of monitoring, the median numbers of days with a record of IMS score, ASMS score, and sleep duration were 5 (IQR 4-8), 4 (IQR 3-4), and 50 (IQR 28-63), respectively. Figure S1 in Multimedia Appendix 1 shows the heatmap of the observation size for each participant in the record of IMS scores, ASMS scores, and sleep duration. It is shown that the records for all the mHealth measures were severely sparse, especially for the IMS scores and ASMS scores, due to poor patient adherence.

To extract the dynamic features of the IMS scores, ASMS scores, and sleep duration from baseline to week 12 for each patient, functional data analysis methods were used [34]. Through functional data analysis methods, the temporally dependent PROs and digital phenotype records have not to be collapsed into a single value, and the temporal dynamic information can be retained [35]. In specific, to adapt to the sparseness of the data caused by poor patient adherence, a method called principal components analysis through conditional expectation (PACE) was used [36]. The PACE approach is a commonly used functional principal component (FPC) analysis method that is designed for sparsely and irregularly observed functional data. This approach uses a local linear smoother for the estimation of the mean function and the covariance function, and then it computes the FPC scores based on conditional expectations. The PACE approach borrows the strength of the entire sample in the computation, so it can make full use of the information from the dataset. By using the fdapace R package with version 0.6.0, we implemented the PACE approach for the records of IMS score, ASMS score, and sleep duration, respectively, and the estimated first FPC scores were treated as the extracted dynamic features.

#### Machine Learning Models

To predict the variation trajectory class of depression severity for the patients, various machine learning methods were used, such as decision tree, random forest, and XGBoost (Extreme Gradient Boosting). In specific, a decision tree completes classification by recursively selecting the best decision rules to partition the data, and the decision paths can be summarized through a tree structure. Random forest and XGBoost are both ensemble learning methods, but with different ensemble patterns. Random forest depends on many independent decision trees, while XGBoost trains a sequence of decision trees in an iterative way. To alleviate the overfit problems for the random forest and XGBoost methods, the node size (minimum size of terminal nodes) was selected as 10 for the random forest model, and the maximum depth of a tree was set as 3 for the XGBoost model. We conducted the above machine learning methods using the rpart, randomForest, and xgboost R packages with versions 4.1.24, 4.7‐1.2, and 1.7.11.1, respectively.

#### Predictive Performance Assessment

To find out how the records of IMS score, ASMS score, and sleep duration contribute to the classification of depression variation, we compared the classification results obtained from different input information. In specific, we considered the prediction based on 4 types of input scenarios. Scenario 1 uses only baseline features; scenario 2 integrates baseline features with the FPC scores of the PROs and digital phenotype records; scenario 3 combines baseline features and HAMD-17 and HAMA scores assessed at week 2; scenario 4 incorporates baseline features, FPC scores of the PROs and digital phenotype records, and HAMD-17 and HAMA scores at week 2. Here, scenario 2 was our central focus, while scenario 1 was used to assess the prediction without mHealth measures. Moreover, scenarios 3 and 4 were introduced to simulate the ideal cases with assessments obtained from clinical visits. By comparing scenario 2 with scenarios 3 and 4, we can shed light on how the predictive performance in scenario 2 approaches the ideal cases.

Moreover, the performance was evaluated by the classification accuracy for the overall data, stable decline patients, and nonstable decline patients. The 95% CIs of the classification accuracy were obtained through the Wilson method based on Hmisc R package. The dataset was randomly split into a training set (80%) and a test set (20%). Given the class imbalance, we randomly oversampled the fluctuate decline, fast decline, and delayed and fluctuate classes to make their class size 80% of the size of the stable decline. The oversampling procedure was implemented within the training set, ensuring a strict separation between the training set and test set.

#### Variable Importance

The importance of the considered predictive factors was assessed for the decision tree, random forest, and XGBoost to illustrate the contribution of mHealth measures in the model construction. Specifically, Gini impurity reduction [37] was used to compute variable importance for the decision tree [38] and random forest models [39], while the Gain metric [40] was used for the XGBoost model. For the implementation, the rpart package, randomForest package, and xgboost package in R were used, respectively. Moreover, we also conducted a permutation test to estimate the significance of variable importance for the random forest model through permuting the response variable 1000 times [41]. A *P* value less than .05 indicates a significant variable contribution. The rfPermute R package with version 2.5.5 was used for the computation.

#### Sensitivity Analysis

Further, to investigate the advantage of the functional data analysis method, we also evaluate the predictive performance when the mean values of IMS score, ASMS score, and sleep duration were used for model construction, rather than their FPC scores. The dynamic features of the PROs and digital phenotype were disrupted because the records were roughly summarized as a single value. Hence, it is hypothesized that using the FPC scores would yield a more accurate prediction.

## Results

### Characteristics of Participants

**Figure 2.**
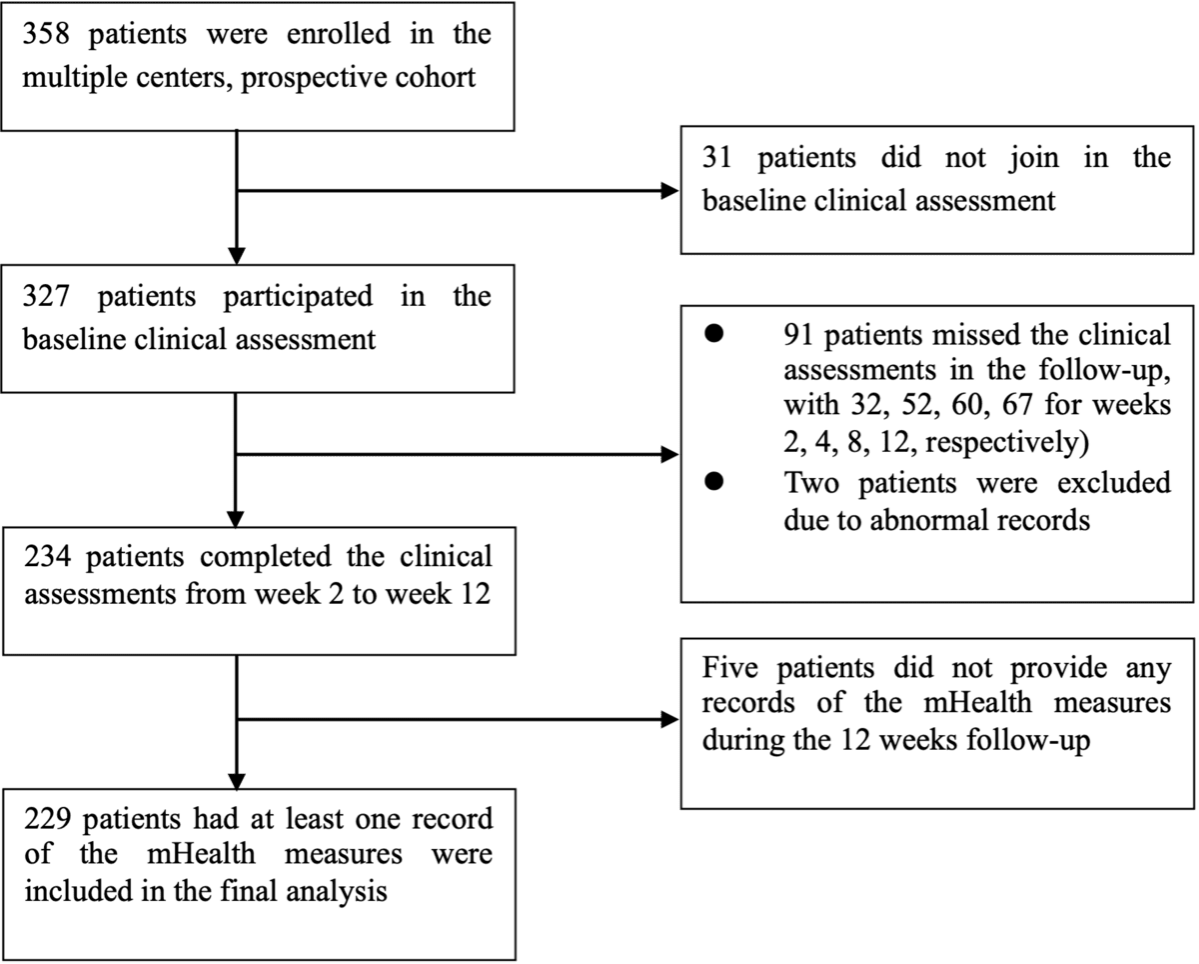
Flowchart illustrating participant inclusion. With 358 patients enrolled in the multiple centers, prospective cohort, 229 patients were finally included in our study. mHealth: mobile health.

A total of 229 patients with MDD were included in the study (Figure 2), with 78 males (34.1%), 151 females (65.9%), and a mean age of 28.7 years. According to the variation of HAMD-17 scores, the participants were labeled as stable decline, fluctuate decline, fast decline, and delayed and fluctuate, respectively. The number of classes was selected as *k*=4 with the consideration of both silhouette coefficients (0.182) and clinical significance. Clinical significance refers to the capacity of the chosen clusters to distinguish 4 clinically recognizable patterns of symptom reduction over 12 weeks, categorizing the longitudinal patient responses into stable decline, fluctuate decline, fast decline, and delayed and fluctuate trajectories.

**Figure 3.**
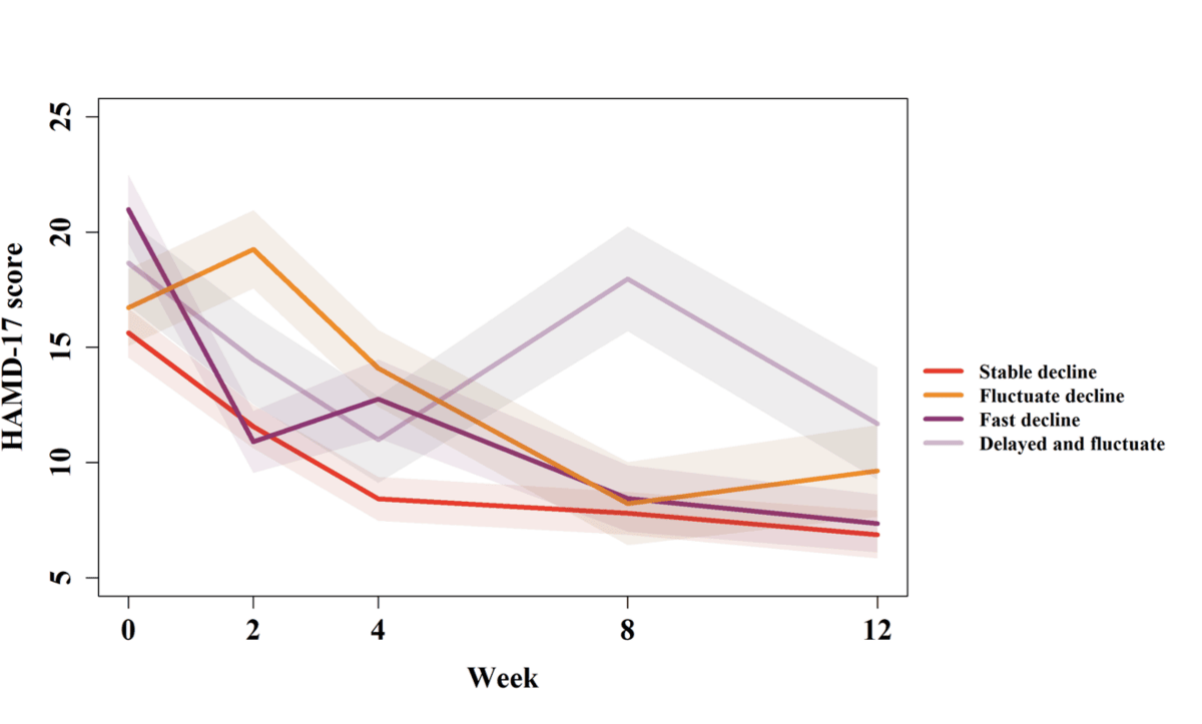
The mean HAMD-17 scores from baseline to week 12 for patients in stable decline, fluctuate decline, fast decline, and delayed and fluctuate classes. HAMD-17: Hamilton Depression Rating Scale.

Figure 3 shows the variation of the mean HAMD-17 scores from baseline to week 12 for each class (refer to Multimedia Appendix 1 for HAMD-17 scores figures when *k*=2,3,5), and Figure S4 in Multimedia Appendix 1 shows that patients within the same class exhibited the same temporal trend of depression severity. In specific, the stable decline class contained 93 (40.6%) patients with MDD, who were more likely to improve steadily during the study. There were 44 (19.2%) patients with MDD in the fluctuate decline class. It is shown that the HAMD-17 scores of participants in the fluctuate decline class might go through an upward fluctuation before week 2 and begin to decrease afterwards. The fast decline class included 60 (26.2%) patients with MDD, and the HAMD-17 scores of these patients showed a rapid decline before week 2. Moreover, 32 (14%) patients with MDD were assigned to the delayed and fluctuate class. It is evident that the HAMD-17 scores of these patients showed a delayed decline and were prone to exhibit a drastic rise at week 8. The symptomatic rebound at week 8 indicates a critical clinical juncture for evaluating treatment efficacy in depression [42]. This pattern suggests a unique phenotype of therapeutic instability where early progress fails to persist. Such findings emphasize the importance of extended clinical monitoring to identify patients at risk of midstage relapse.

Table 1 reports the demographic and clinical characteristics of the patients with MDD in each class. It can be observed that the HAMD-17 and HAMA scores, as well as the changes in HAMD-17 scores, were significantly different among these 4 classes for each week. In addition, the average IMS scores during the follow-up were also significantly different among these 4 classes. Moreover, there was no significant difference in sex, age, BMI, and baseline Quality of Life Enjoyment and Satisfaction Questionnaire—Short Form, Sheehan Disability Scale, IMS, and ASMS scores among these 4 classes. Refer to Multimedia Appendix 1 for the figures of mean curves of IMS scores, ASMS scores, and sleep duration for each class, and for the demographic characteristics of patients in each participating center.

**Table 1.** Demographic and clinical characteristics of patients with major depressive disorder in the study.

Features	All (n=229)	Stable decline (n=93)	Fluctuate decline (n=44)	Fast decline (n=60)	Delayed and fluctuate (n=32)	Statistics^a^ (*df*=3)	*P* value
Sex, n (%)						1.74	.63
Male	78 (34.1)	29 (31.2)	17 (38.6)	23 (38.3)	9 (28.1)		
Female	151 (65.9)	64 (68.8)	27 (61.4)	37 (61.7)	23 (71.9)		
Age (years), mean (SD)	28.7 (8.9)	30.4 (10.2)	27.0 (7.4)	28.4 (8.4)	27.0 (6.3)	3.73	.29
Baseline BMI, mean (SD)	22.2 (3.9)	22.3 (4.0)	21.8 (3.9)	22.2 (3.7)	22.6 (3.9)	1.25	.74
Baseline HAMD-17^b^, mean (SD)	17.7 (5.9)	15.6 (5.2)	16.7 (5.7)	21.0 (5.9)	18.7 (5.3)	28.33	<.001
Baseline HAMA^c^, mean (SD)	15.6 (6.7)	13.8 (5.5)	15.8 (7.0)	17.8 (7.6)	16.7 (6.5)	10.84	.01
Baseline Q-LES-Q-SF^d^, mean (SD)	44.9 (9.4)	46.1 (10.1)	43.1 (7.9)	44.9 (9.7)	43.8 (8.9)	4.65	.20
Baseline SDS^e^, mean (SD)	13.9 (6.5)	13.0 (6.7)	13.5 (5.4)	14.6 (6.8)	15.4 (6.6)	4.13	.25
Work/school, mean (SD)	5.0 (2.7)	4.9 (2.8)	4.8 (2.5)	5.1 (2.7)	5.3 (2.8)	1.21	.75
Social life/leisure activities, mean (SD)	4.8 (2.5)	4.4 (2.6)	4.7 (2.2)	5.2 (2.5)	5.2 (2.3)	4.75	.19
Family life/home responsibilities, mean (SD)	4.1 (2.6)	3.8 (2.7)	4.0 (2.3)	4.3 (2.8)	4.9 (2.4)	5.46	.14
Baseline IMS^f^, mean (SD)	71.9 (25.8)	73.4 (24.5)	64.0 (24.1)	76.3 (29.3)	70.1 (23.0)	6.01	.11
Baseline ASMS^g^, mean (SD)	2.7 (2.3)	2.8 (2.4)	2.1 (1.6)	3.1 (2.4)	2.1 (2.2)	7.02	.07
HAMD-17 at week 2, mean (SD)	13.3 (6.0)	11.6 (4.6)	19.2 (5.7)	10.9 (5.3)	14.5 (5.6)	54.45	<.001
HAMD-17 at week 4, mean (SD)	11.0 (6.0)	8.4 (4.7)	14.1 (5.6)	12.8 (6.7)	11.0 (5.4)	35.46	<.001
HAMD-17 at week 8, mean (SD)	9.5 (6.4)	7.8 (4.6)	8.2 (6.1)	8.4 (5.7)	18.0 (6.6)	47.51	<.001
HAMD-17 at week 12, mean (SD)	8.2 (5.9)	6.9 (5.0)	9.6 (6.7)	7.4 (5.0)	11.7 (7.0)	15.91	.001
HAMD-17 changes up to week 2, mean (SD)	4.4 (5.6)	4.1 (3.1)	–2.5 (3.7)	10.1 (4.1)	4.2 (4.6)	133.71	<.001
HAMD-17 changes up to week 4, mean (SD)	6.7 (6.0)	7.2 (4.8)	2.6 (5.8)	8.2 (7.0)	7.7 (5.3)	26.22	<.001
HAMD-17 changes up to week 8, mean (SD)	8.2 (6.8)	7.8 (5.1)	8.5 (7.4)	12.5 (5.7)	0.7 (5.5)	63.66	<.001
HAMD-17 changes up to week 12, mean (SD)	9.5 (6.7)	8.7 (5.5)	7.1 (7.7)	13.6 (5.8)	7.0 (6.5)	34.21	<.001
HAMA at week 2, mean (SD)	11.9 (6.7)	10.5 (5.5)	16.3 (7.0)	10.3 (6.4)	13.1 (7.4)	25.21	<.001
HAMA at week 4, mean (SD)	10.5 (6.3)	8.3 (5.2)	13.3 (6.2)	11.8 (6.8)	10.3 (6.3)	23.89	<.001
HAMA at week 8, mean (SD)	8.6 (6.6)	7.0 (5.3)	7.2 (5.5)	8.2 (5.5)	15.6 (8.4)	29.51	<.001
HAMA at week 12, mean (SD)	7.8 (6.4)	6.8 (5.5)	9.1 (7.9)	6.9 (5.4)	10.8 (7.2)	10.31	.02
Average IMS during follow-up, mean (SD)	76.8 (18.8)	79.6 (16.2)	70.9 (15.0)	79.4 (22.0)	72.0 (22.2)	12.29	.006
Average ASMS during follow-up, mean (SD)	3.3 (1.9)	3.5 (1.8)	2.9 (1.5)	3.5 (2.3)	2.8 (1.8)	4.77	.19

a Chi-square test for sex and Kruskal-Wallis rank sum test for other features.

b HAMD-17: Hamilton Depression Rating Scale.

c HAMA: Hamilton Anxiety Scale.

d Q-LES-Q-SF: Quality of Life Enjoyment and Satisfaction Questionnaire—Short Form.

e SDS: Sheehan Disability Scale.

f IMS: Immediate Mood Scaler.

g ASMS: Altman Self-Rating Mania Scale.

### Prediction of Depression Variation Pattern With Digital mHealth Measures

**Figure 4.**
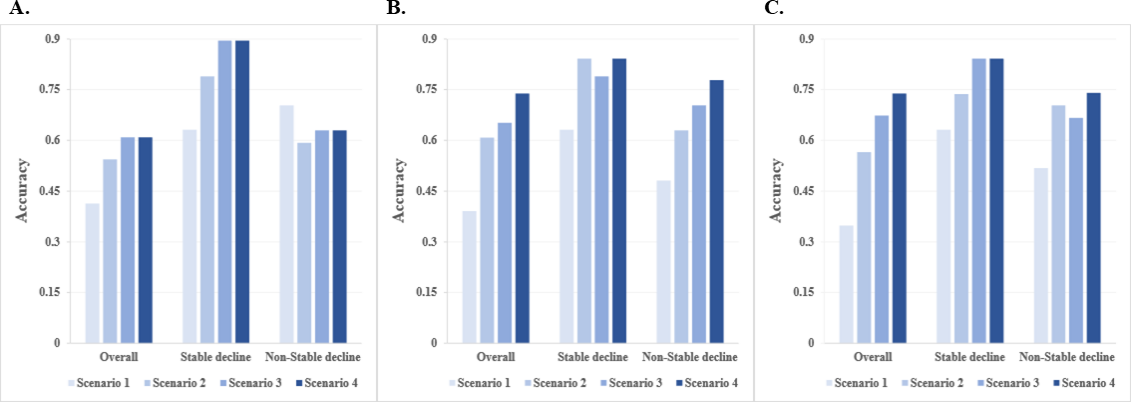
Classification accuracy for the overall data, stable decline patients, and nonstable decline patients with different input information based on the machine learning algorithms: (A) decision tree, (B) random forest, and (C) XGBoost. Scenario 1: baseline features; scenario 2: baseline features and FPC scores of the PROs and digital phenotype records; scenario 3: baseline features and HAMD-17 and HAMA scores at week 2; scenario 4: baseline features, FPC scores of the PROs and digital phenotype records, and HAMD-17 and HAMA scores at week 2. FPC: functional principal component; HAMA: Hamilton Anxiety Scale; HAMD-17: Hamilton Depression Rating Scale; PRO: patient-reported outcome; XGBoost: Extreme Gradient Boosting.

Figure 4 and Table S2 in Multimedia Appendix 1 show the classification results obtained from various input information by different machine learning approaches. It is shown that by using baseline features and FPC scores of the PROs and digital phenotype records (scenario 2), decision tree, random forest, and XGBoost all give reasonable classification accuracy for the overall data (54.35%, 60.87%, and 56.52%), stable decline patients (78.95%, 84.21%, and 73.68%), and nonstable decline patients (59.26%, 62.96%, and 70.37%). On the other hand, when the models were constructed without dynamic features of the digital mHealth measures (scenario 1), all considered machine learning approaches show poor classification efficacy, which indicates the great importance of digital mHealth measures in the monitoring of depression variation. Further, comparing the predictive performance in scenario 2 with that in scenarios 3 and 4, though the classification accuracy was elevated, the improvement was not as obvious as that achieved by changing from scenario 1 to scenario 2. Moreover, despite a marginal reduction compared to the ideal clinical scenarios, scenario 2 offers practical advantages in terms of scalability and reduces assessment burden for continuous follow-ups.

### Contribution of Digital mHealth Measures

Figure S5 and Table S3 in Multimedia Appendix 1 show the variable importance for decision tree, random forest, and XGBoost in the prediction of depression variation patterns using baseline features and FPC scores of the PROs and digital phenotype records (scenario 2). For both random forest and XGBoost, which yielded more accurate classification, the dynamic feature of IMS score records was of paramount importance in the model construction. Moreover, FPC scores of the ASMS score and sleep duration also played a dominant role in the modeling, closely following the baseline HAMD-17 score. Table S4 in Multimedia Appendix 1 reports the *P* values of the permutation test, which confirm the significance of the contribution of both baseline HAMD-17 score and mHealth measures. The variable importance results demonstrated the great contribution of digital mHealth measures in the prediction of depression variation.

### Sensitivity Analysis

Table S5 in Multimedia Appendix 1 reports the classification results of decision tree, random forest, and XGBoost with the use of baseline features and the mean values of IMS score, ASMS score, and sleep duration, rather than the FPC scores of them (scenario 2). Compared with introducing the dynamic features of the mHealth measures, using the mean values led to inferior prediction, especially for decision trees (overall data: 54.35% vs 47.83%; stable decline: 78.95% vs 47.37%; nonstable decline: 59.26% vs 59.26%) and random forests (overall data: 60.87% vs 50.00%; stable decline: 84.21% vs 78.95%; nonstable decline: 62.96% vs 55.56%). The results implied the necessity of considering the temporal dynamics of the digital mHealth measures.

## Discussion

### Principal Findings

This study evaluated the contribution of digital mHealth measures, including PROs and digital phenotype, to the prediction of the variation of depression within 12 weeks for patients with MDD. Based on the variation of HAMD-17 scores at 5 visits within 12 weeks, each patient was labeled as stable decline, fluctuate decline, fast decline, or delayed and fluctuate. The study used functional data analysis methods to address the severe sparsity of PROs and digital phenotype records caused by poor patient adherence, thereby effectively extracting their dynamic features. The classification results showed that by combining the baseline features with the dynamic information of PROs and digital phenotypes, the depression variation pattern can be identified with reasonable accuracy. Overall, our study demonstrated that the dynamic features of mHealth measures extracted by the functional data analysis method can be an effective alternative to clinical visits for depression variation monitoring. The findings can alleviate the burdens for both patients and doctors.

For the detection of latent trajectory classes regarding depression severity, growth mixture modeling was widely used in the previous studies [43-45]. However, growth mixture modeling was more likely to capture the similarity in the magnitude of depression severity, rather than its changing pattern. In our study, the patients with MDD were classified into 4 classes based on *k*-means clustering through the HAMD-17 score differences. Within each class, the patients exhibited the same temporal trend of depression severity. The classes were further named based on the dynamic characteristics of the depression symptom trajectories. In specific, stable decline implies a stable improvement of depression symptoms, fluctuate decline indicates a fluctuation of depression symptoms in the early stage and an alleviation at the end, fast decline represents a high baseline severity and a rapid improvement of depression symptoms, while delayed and fluctuate means a delayed improvement and sharp variation of depression symptoms.

Our findings revealed that the dynamic features of digital mHealth measures can reflect the depression variation of patients with MDD. By recording the trajectories of IMS scores, ASMS scores, and sleep duration using the smartphone and wristband, the dynamic characteristics of depression severity can be evaluated without strong dependence on continuous tracking of HAMD-17 scores. Moreover, results from the variable importance analysis highlighted the contribution of the mHealth measures in the depression variation prediction.

Further, the sensitivity analysis indicated that taking into account the temporal dynamics of the mHealth measures is beneficial. In our study, the dynamic features of PROs and digital phenotypes were fully characterized through a functional data analysis method, rather than aggregating the records to a single scalar summary measure (eg, mean or median) by collapsing over time. Functional data analysis is popular in the study of continuously recorded data, such as physical activity data [46] and electroencephalography data [35], which supports the use of functional data analysis methods in our research. However, classical functional data analysis methods are only suitable for densely collected data. To extract dynamic features of the sparsely collected PROs and digital phenotypes, the PACE method was applied, which is a commonly used method for the principal component analysis of sparsely and irregularly observed functional data [36]. Collectively, the benefits of using functional data analysis methods can be summarized in two aspects: (1) avoiding the loss of information related to temporal dynamics of the PROs and digital phenotype records and (2) conquering the problem of sparse observations caused by poor patient adherence without excluding any participants with a low observation size (or in other words, with a high percentage of missing observations).

### Comparison With Previous Work

Previous studies have demonstrated the potential of mHealth measures in predicting symptom severity of patients with MDD [6,23,47]. To further monitor the depression stability, Bai et al [20] defined two types of variation patterns, steady and swing, based on the patient-reported Patient Health Questionnaire-9 scores. However, such PRO records cannot substitute for the gold-standard HAMD-17 scores evaluated by clinicians in the longitudinal monitoring of patients with MDD. In our study, the more credible clinician-reported scores were used for outcome labeling, and PROs were incorporated as predictive factors, underscoring the need for more sophisticated use of PRO records. Moreover, Ikäheimonen et al [48] also considered variation of depression by partitioning the participants into 4 classes: declines, increases, remains depressed, and remains nondepressed. Although their model based on smartphone behavioral data achieved an overall accuracy of 75%, the predicting efficacy for classes with state changes (declines and increases) was invalid with a precision lower than 50%. Compared with their work, we refined the depression variation labels to facilitate the detection of specific temporal patterns, and our model yielded a more robust predictive performance for patients with fluctuating clinical courses. This improvement likely stems from our application of the functional data analysis to the mHealth records, which can effectively characterize the continuous underlying transition patterns that traditional summary statistics might overlook.

The core contribution of our study lies in the methodological integration of dual temporal dynamics for depression severity and mHealth measures, and the capacity to handle extremely sparse mHealth records. First, our definition of the outcome labels effectively characterized the temporal evolution of depression severity, enabling the identification of the inherent heterogeneity in depression progression over time. Moreover, the dynamic features of mHealth measures were extracted to predict the changing patterns of depression trajectory, forming an innovative dual-dynamic framework that is more practical in real-world psychiatric monitoring. Last but not least, our study made it feasible to obtain an acceptable depression monitoring using mHealth records with great irregularity and sparsity, which significantly enhances the ecological validity of our model and mitigates the adherence-related barriers that hinder clinical application of mHealth measures.

In recent years, high-resource biological markers derived from neuroimaging [49] and electrophysiology [50] have gained prominence in predicting antidepressant response due to their insights into underlying mechanisms. Compared with these markers, low-cost mHealth measures possess the ability of high-frequency, continuous recording within a naturalistic environment, facilitating scalable real-world monitoring and the dynamic capture of longitudinal symptom variations that static biomarkers may overlook. Given the complementary strengths, the integration of high-resource biological markers with real-world mHealth data represents a promising avenue for personalized psychiatry that warrants further investigation.

### Strengths and Limitations

The strengths of our study can be summarized as follows. First, this study contained a long period of continuous monitoring for a large number of patients with MDD. Second, our study overcame the missing and sparse problems that universally existed in natural observations with the use of functional data analysis methods, which also facilitated the characterization of the temporal dynamics of PROs and digital phenotype records. Third, our study effectively reduced the dependence of patient adherence and eased the heavy burden of clinical follow-up in the prediction of depression variation trajectory.

Our study still has several limitations. One limitation is that only the IMS score, ASMS score, and sleep duration were continuously monitored and were used in the prediction of depression severity. Additional collection of more indicators of PROs and digital phenotype would be beneficial [27]. The other limitation is that only HAMD-17 scores were used in the construction of the labels of depression variation, which may be insufficient. Therefore, collecting more indicators of depressive symptoms and putting them together would be advantageous for the characterization of the change of depression severity. In addition, as the entire sample was used in the *k*-means clustering analysis to ensure a robust definition of clinical phenotypic labels, an implicit data leakage was introduced into the model assessment. So, the results, which may be inclined toward optimism, should be interpreted with caution. Moreover, constrained by the limited and imbalanced sample sizes of the participating centers, the predictive performance exhibited variability across heterogeneous clinical environments. This suboptimal performance underscores the inherent challenges of model generalizability. Thus, further research is required to enhance the robustness of the model when applied to diverse clinical settings. Last, while the predicted depression variation pattern can guide further treatment for the patient with MDD, it was not able to provide an earlier warning within 12 weeks, which is worth further research.

### Conclusions

Our study demonstrated the potential of mHealth measures, as an alternative to clinical visits, in the monitoring of depression variation, even under poor patient adherence. The results suggested that dynamic features of PROs and digital phenotypes, combined with baseline clinical features, can provide clinically meaningful information for depression variation patterns. Our findings help reduce dependence on strict patient adherence and alleviate the burdens of frequent clinical follow-ups, which facilitates a more practical monitoring of depression trajectory variations.

## Supplementary material

10.2196/81397 Multimedia Appendix 1 Additional statistical results.

## Abbreviations

ASMS: Altman Self-Rating Mania Scale
FPC: functional principal component
HAMA: Hamilton Anxiety Scale
HAMD-17: Hamilton Depression Rating Scale
IMS: Immediate Mood Scaler
MDD: major depressive disorder
mHealth: mobile health
PACE: principal components analysis through conditional expectation
PRO: patient-reported outcome
