# Supplementary material for: Contribution of Longitudinal Mobile Health Measures in the Dynamic Track of Patients With Major Depressive Disorder: Multiple Centers, Prospective Cohort Study Using Functional Data Analysis and Machine Learning
Source: JMIR Mhealth Uhealth. 2026 May 11;14:e81397. doi: 10.2196/81397 (PMC13160482; doi:10.2196/81397)
Supplement: Multimedia Appendix 1 [file mhealth-v14-e81397-s001.docx]

## **Multimedia Appendix 1: Additional Statistical Results**

### **Heatmap of the Observation Size for Each Participant**

Figure S1. Heatmap of the observation size for each participant in the record of the mHealth measures. Figure S1a. IMS scores; Figure S1b. ASMS scores; Figure S1c. sleep duration.


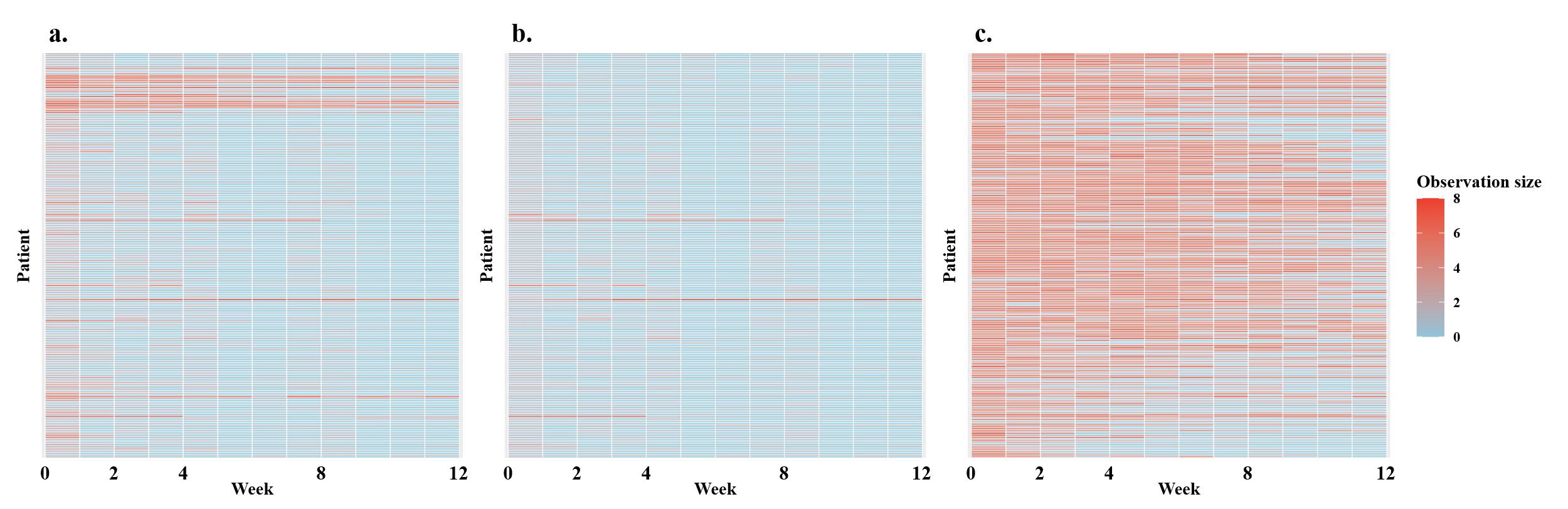


### **Mean Curves of IMS Scores, ASMS Scores and Sleep Duration**

Figure S2 shows the mean curves of IMS scores, ASMS scores and sleep duration for the overall data and the four classes. It can be observed that the mean IMS score curve showed an increasing trend within the 12 weeks, while the mean ASMS score rose first, and began to decrease after week 8. From week 2 to week 8, the mean sleep duration went up with fluctuations.

Figure S2. The mean curves of IMS scores, ASMS scores and sleep duration for the overall data and the four classes (Stable decline, Fluctuate decline, Fast decline, Delayed and fluctuate).


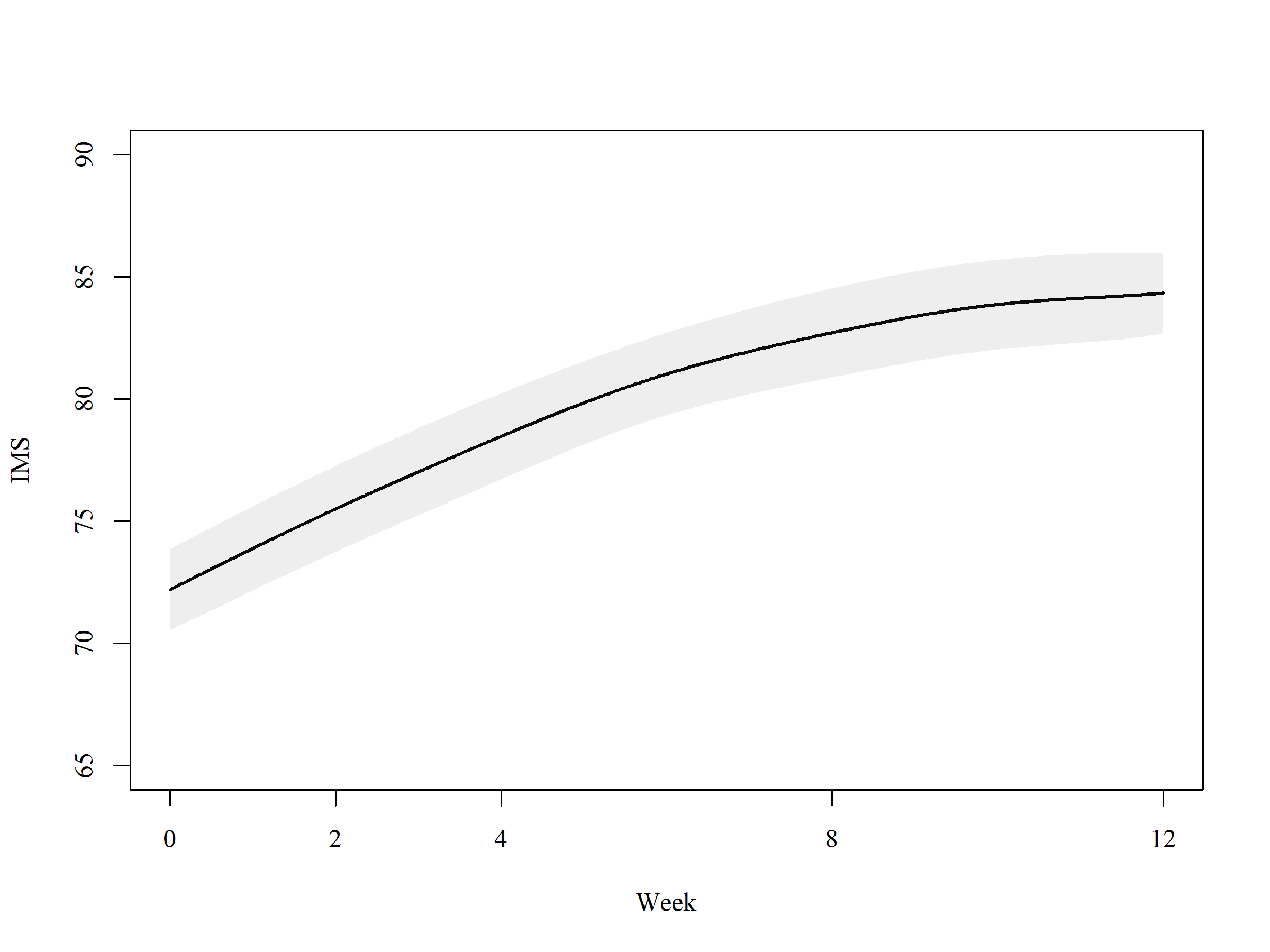

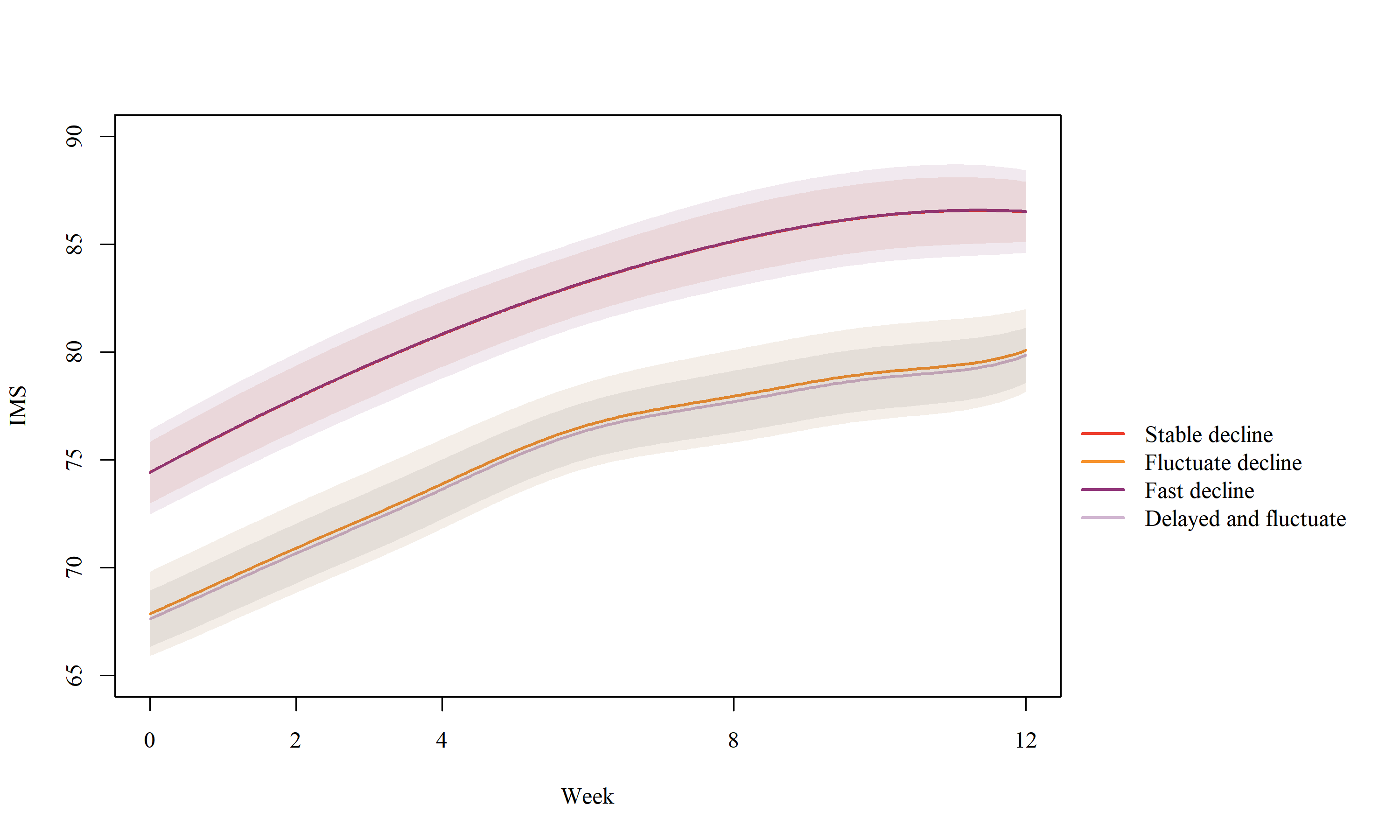


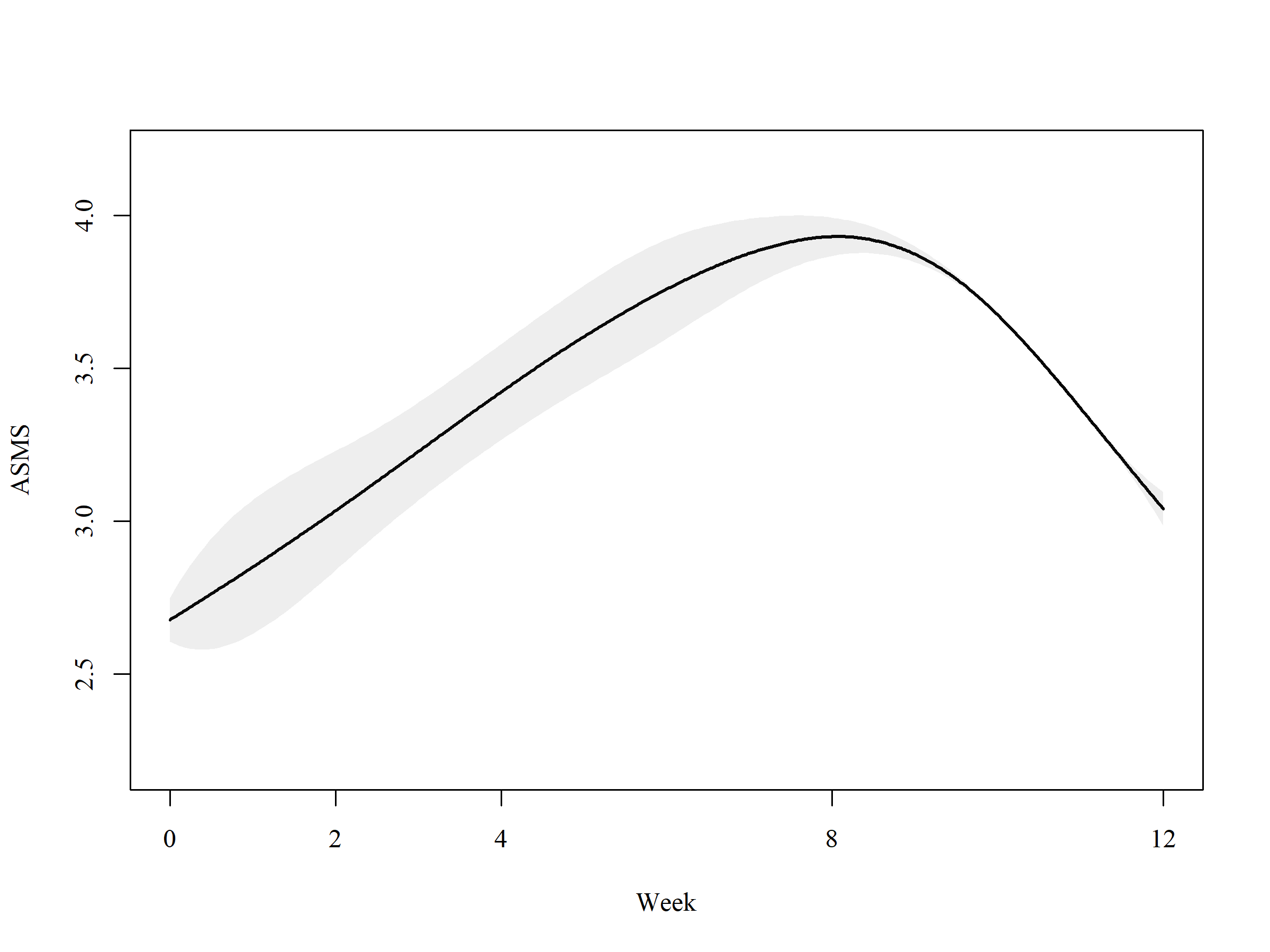

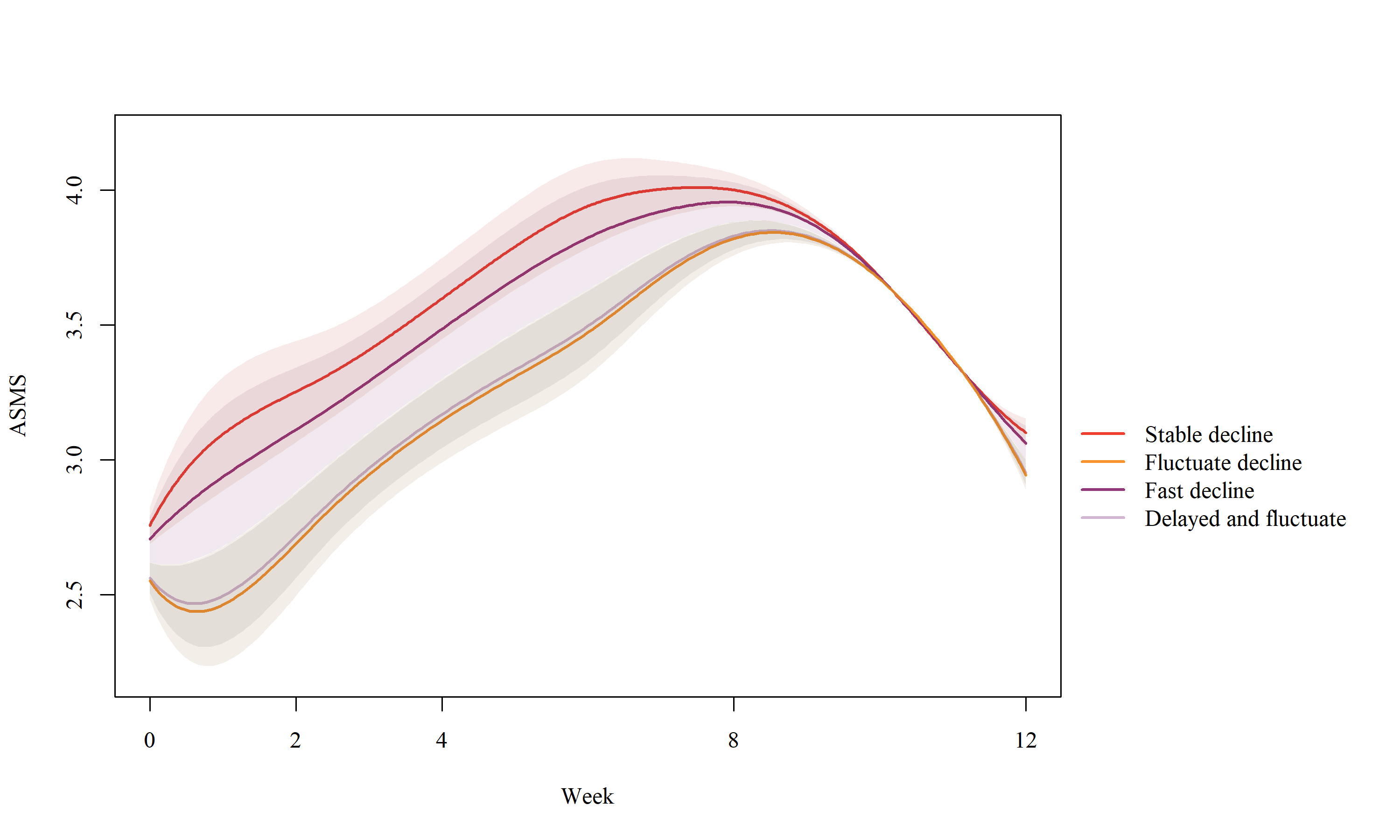


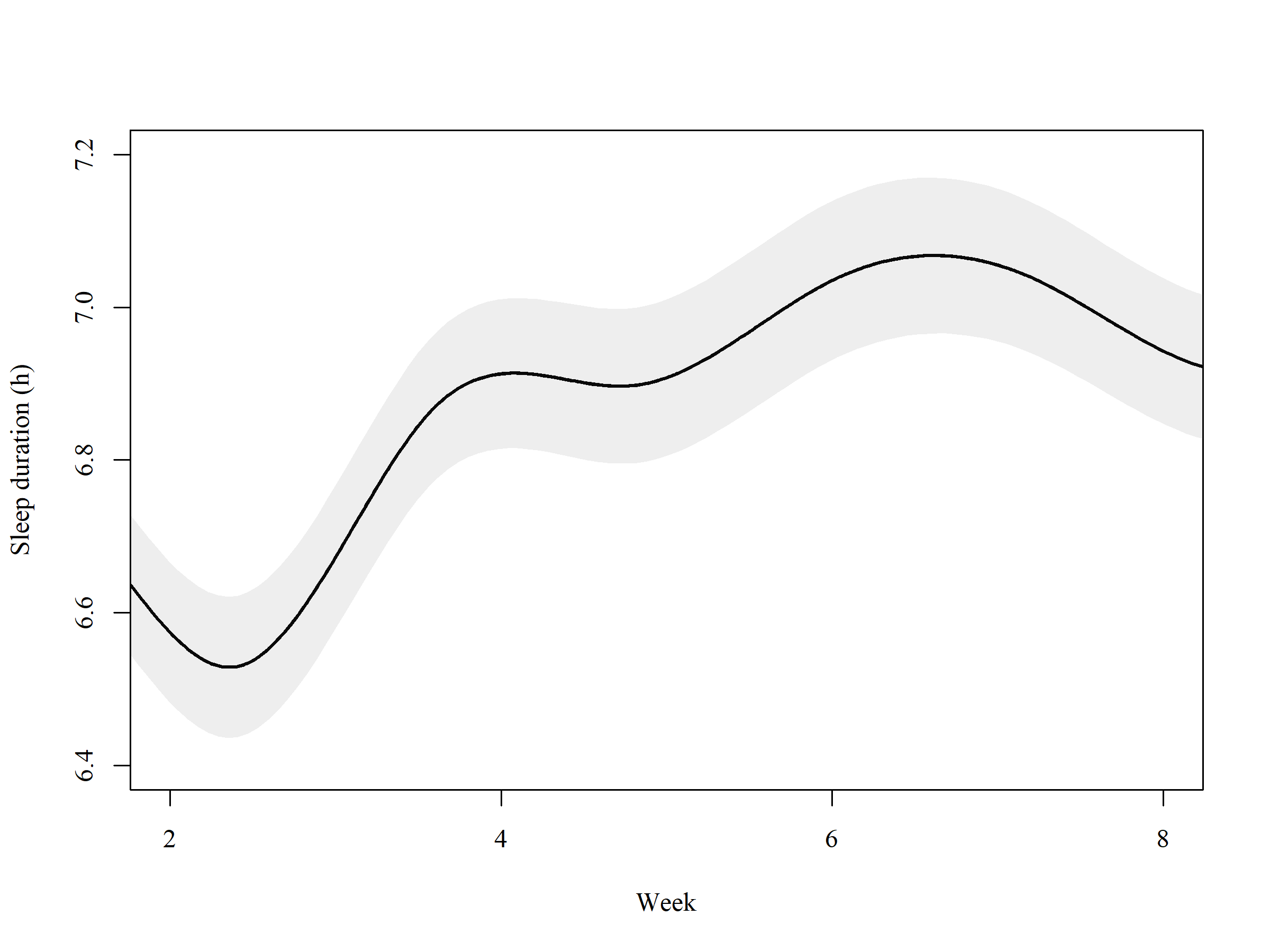

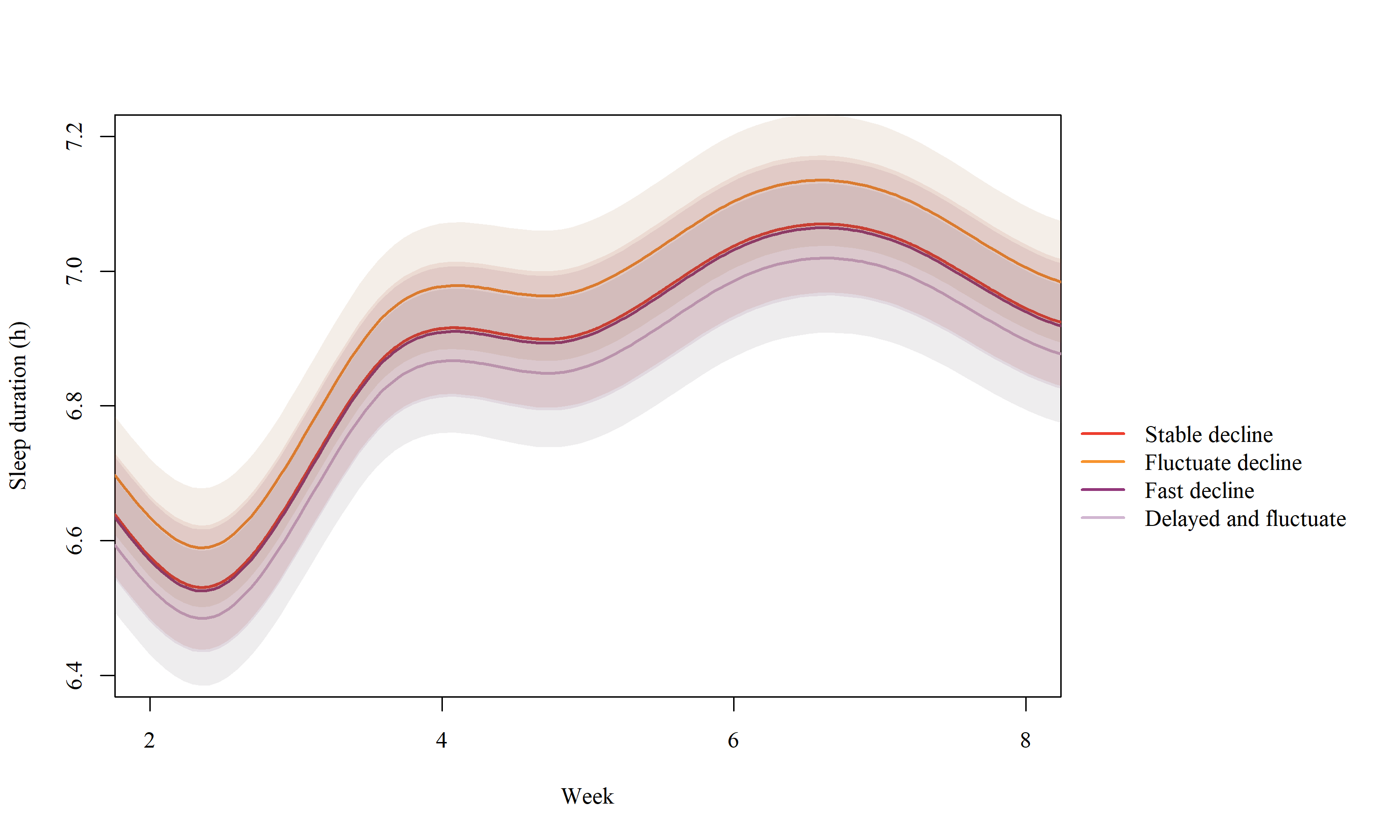


### **Additional Clustering Results**

Figure S3. The mean HAMD-17 scores from baseline to week 12 for each cluster when $k=2, 3, 5$.


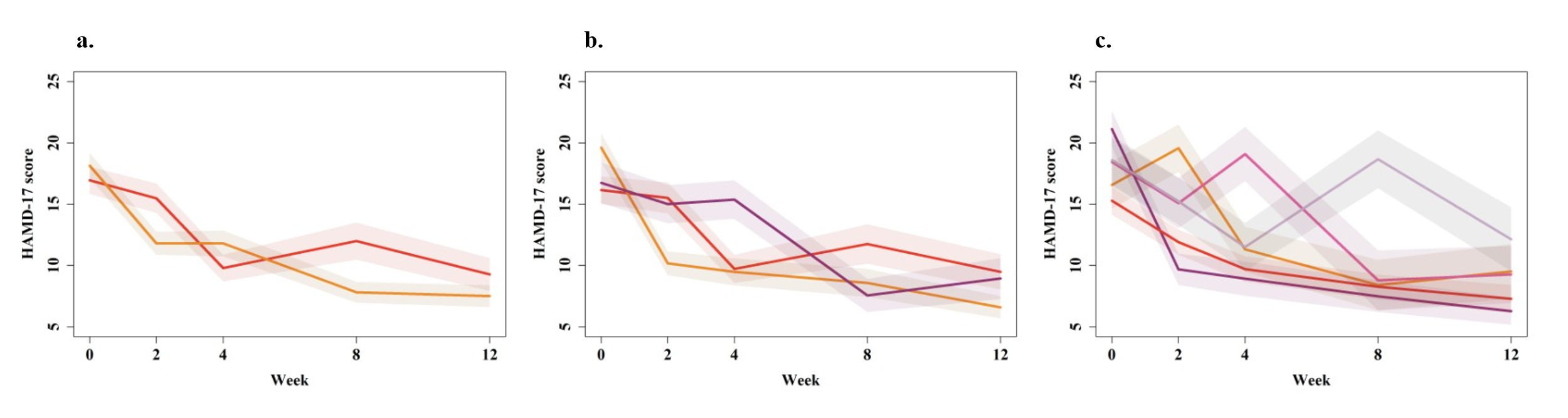


Figure S4. The trajectory of HAMD-17 scores from baseline to week 12 for each participant in the four depression variation classes obtained by k-means clustering: Figure S4a. Stable decline; Figure S4b. Fluctuate decline; Figure S4c. Fast decline; Figure S4d. Delayed and fluctuate.


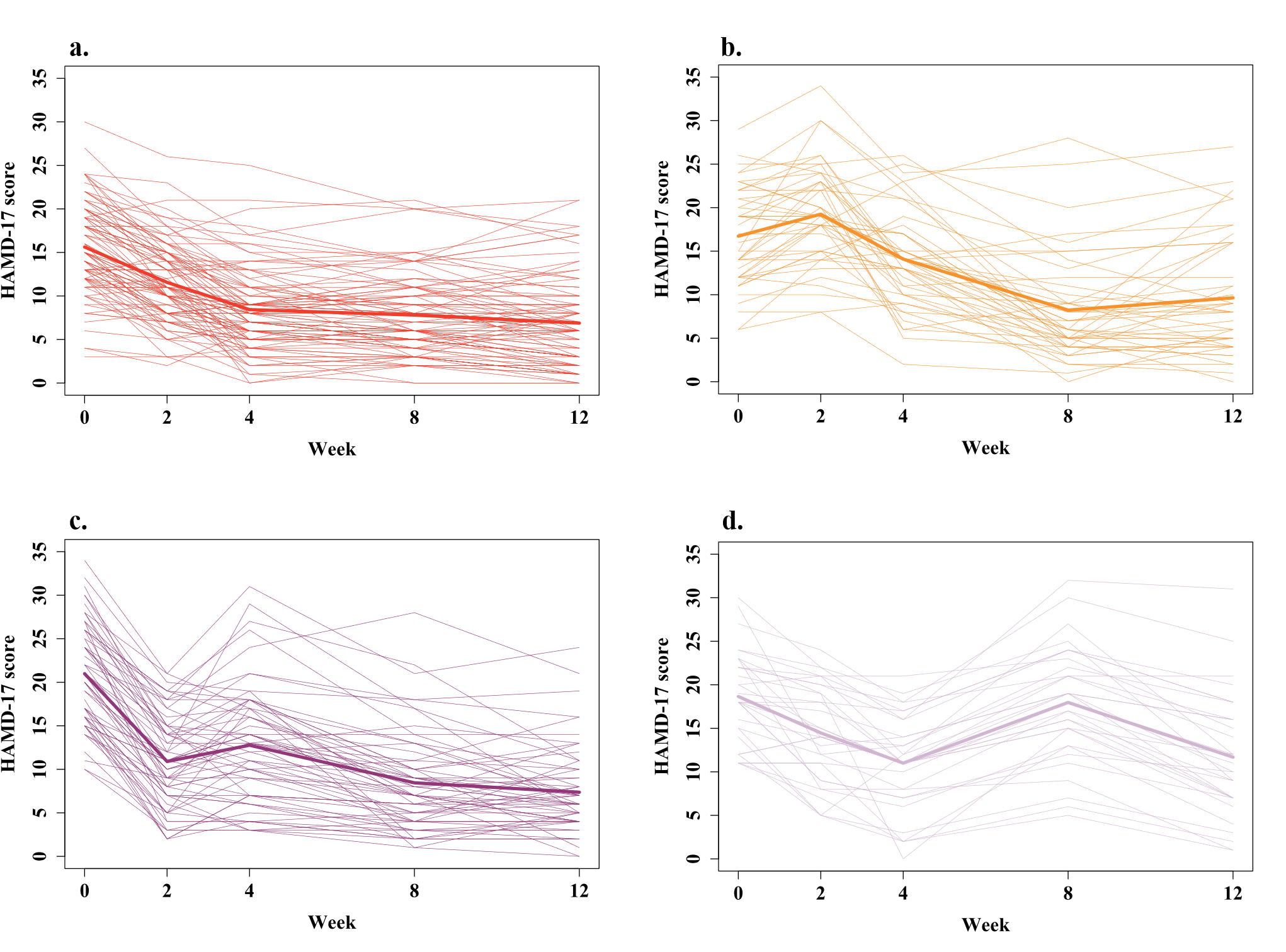


### **Demographic Characteristics of Patients in different participating centers**

Table S1. Demographic Characteristics of MDD patients in each participating center.

| Features | All  (n = 229) | Center 1  (n = 195) | Center 2  (n = 18) | Center 3  (n = 4) | Center 4  (n = 12) | Statistics^a^  (df = 3) | *P* value |
| --- | --- | --- | --- | --- | --- | --- | --- |
| Sex, N (%) |  |  |  |  |  |  |  |
| Male | 78 (34.1) | 67 (34.4) | 6 (33.3) | 2 (50.0) | 3 (25.0) | 0.90 | 0.82 |
| Female | 151 (65.9) | 128 (65.6) | 12 (66.7) | 2 (50.0) | 9 (75.0) |  |  |
| Age, mean (SD), years | 28.7 (8.9) | 28.3 (8.6) | 28.2 (11.2) | 34.2 (8.7) | 34.6 (7.3) | 11.17 | 0.01 |
| Baseline BMI, mean (SD) | 22.2 (3.9) | 22.4 (3.9) | 20.8 (1.9) | 23.7 (4.2) | 20.4 (3.8) | 7.09 | 0.07 |

^a^Statistics: Chi-square test for sex and Kruskal-Wallis rank sum test for other features.

### **Classification Results Obtained from Various Input Information by Different Machine Learning Approaches**

Table S2. Classification accuracy for the overall data, Stable decline patients and non-Stable decline patients obtained by decision tree, random forest and XGBoost, with 95% confidence interval in parentheses.

|  | Overall data | Stable decline | Non-Stable decline |
| --- | --- | --- | --- |
| Decision tree |  |  |  |
| Scenario 1^a^ | 0.4130 (0.2829, 0.5566) | 0.6316 (0.4104, 0.8085) | 0.7037 (0.5152, 0.8415) |
| Scenario 2^b^ | 0.5435 (0.4018, 0.6785) | 0.7895 (0.5667, 0.9149) | 0.5926 (0.4073, 0.7549) |
| Scenario 3^c^ | 0.6087 (0.4646, 0.7361) | 0.8947 (0.6861, 0.9706) | 0.6296 (0.4423, 0.7847) |
| Scenario 4^d^ | 0.6087 (0.4646, 0.7361) | 0.8947 (0.6861, 0.9706) | 0.6296 (0.4423, 0.7847) |
| Random Forest |  |  |  |
| Scenario 1 | 0.3913 (0.2639, 0.5354) | 0.6316 (0.4104, 0.8085) | 0.4815 (0.3074, 0.6601) |
| Scenario 2 | 0.6087 (0.4646, 0.7361) | 0.8421 (0.6243, 0.9448) | 0.6296 (0.4423, 0.7847) |
| Scenario 3 | 0.6522 (0.5077, 0.7732) | 0.7895 (0.5667, 0.9149) | 0.7037 (0.5152, 0.8415) |
| Scenario 4 | 0.7391 (0.5974, 0.8440) | 0.8421 (0.6243, 0.9448) | 0.7778 (0.5924, 0.8939) |
| XGBoost |  |  |  |
| Scenario 1 | 0.3478 (0.2268, 0.4923) | 0.6316 (0.4104, 0.8085) | 0.5185 (0.3399, 0.6926) |
| Scenario 2 | 0.5652 (0.4225, 0.6979) | 0.7368 (0.5121, 0.8819) | 0.7037 (0.5152, 0.8415) |
| Scenario 3 | 0.6739 (0.5297, 0.7913) | 0.8421 (0.6243, 0.9448) | 0.6667 (0.4782, 0.8136) |
| Scenario 4 | 0.7391 (0.5974, 0.8440) | 0.8421 (0.6243, 0.9448) | 0.7407 (0.5532, 0.8683) |

^a^Scenario 1: Baseline features.

^b^Scenario 2: Baseline features and FPC scores of the PROs and digital phenotype records.

^c^Scenario 3: Baseline features and HAMD-17 and HAMA scores at week 2.

^d^Scenario 4: Baseline features, FPC scores of the PROs and digital phenotype records, and HAMD-17 and HAMA scores at week 2

### **Variable Importance for Prediction of Depression Variation Pattern**

Table S3. The variable importance for the prediction of depression variation pattern using baseline features and FPC scores of the PROs and digital phenotype records (scenario 2), based on decision tree, random forest and XGBoost.

| Decision tree | | Random forest | | XGBoost | |
| --- | --- | --- | --- | --- | --- |
| Variables | Importance | Variables | Importance | Variables | Importance |
| HAMD_wk0 | 15.90 | IMS | 18.55 | IMS | 0.20 |
| IMS | 15.21 | HAMD_wk0 | 17.07 | HAMD_wk0 | 0.18 |
| ASMS | 8.56 | ASMS | 16.52 | ASMS | 0.17 |
| Age | 7.22 | Sleep | 16.40 | Sleep | 0.16 |
| HAMA_wk0 | 4.84 | Age | 15.35 | Age | 0.15 |
| Sleep | 3.02 | HAMA_wk0 | 13.06 | HAMA_wk0 | 0.09 |
| Family | 1.16 | Family | 8.59 | Family | 0.03 |
| Sex | 0 | Sex | 2.71 | Sex | 0.02 |

Figure S5. The variable importance for the prediction of depression variation pattern using baseline features and FPC scores of the PROs and digital phenotype records (scenario 2), based on the machine learning algorithms: (a) Decision tree, (b) Random forest, (c) XGBoost.


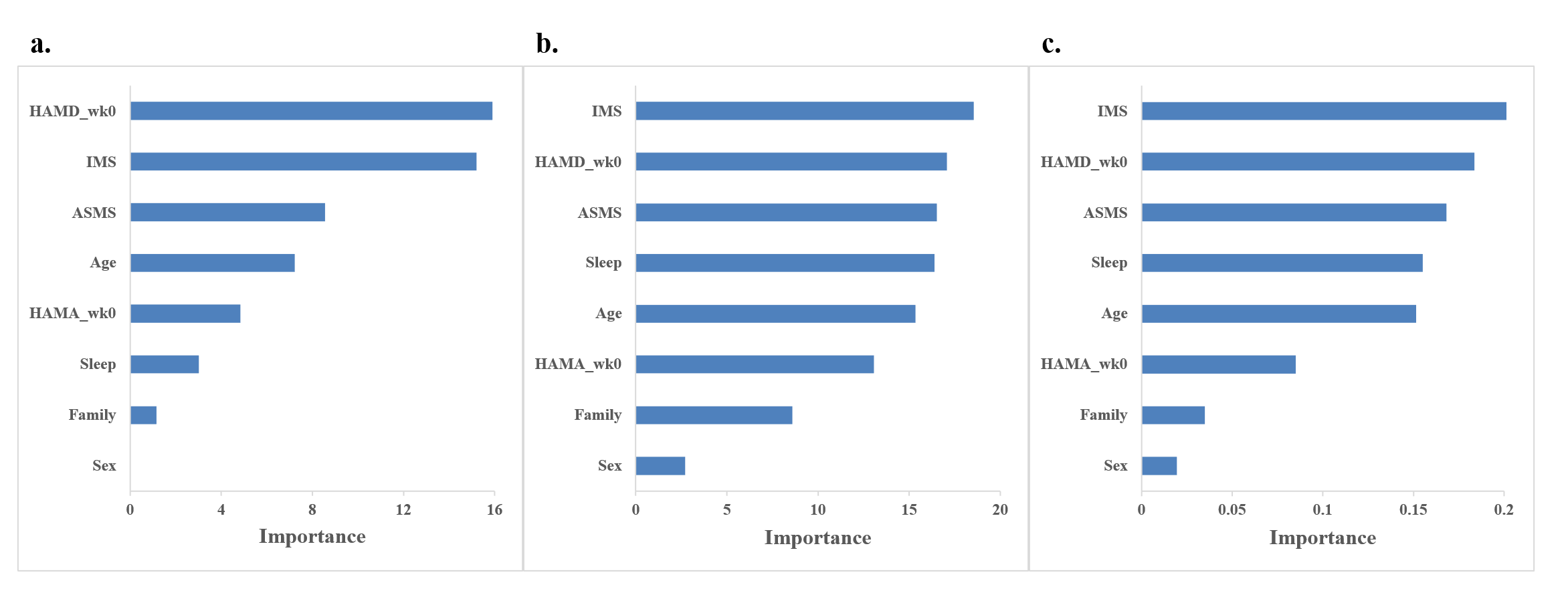


Table S4. Permutation test results of variable importance for random forest model.

| Variables | *P* values |
| --- | --- |
| IMS | <.001 |
| HAMD_wk0 | <.001 |
| ASMS | .005 |
| Sleep | .004 |
| Age | <.001 |
| HAMA_wk0 | .008 |
| Family | .24 |
| Sex | .07 |

### **Sensitivity Analysis**

Table S5. Classification accuracy for the overall data, Stable decline patients and non-Stable decline patients with the use of baseline features and the mean values of IMS score, ASMS score and sleep duration.

|  | Overall data | Stable decline | Non-stable decline |
| --- | --- | --- | --- |
| Decision tree | 0.4783 (0.3412, 0.6186) | 0.4737 (0.2733, 0.6829) | 0.5926 (0.4073, 0.7549) |
| Random forest | 0.5000 (0.3612, 0.6388) | 0.7895 (0.5667, 0.9149) | 0.5556 (0.3731, 0.7241) |
| XGBoost | 0.5652 (0.4225, 0.6979) | 0.7895 (0.5667, 0.9149) | 0.6667 (0.4782, 0.8136) |
